# The Effects of *Calligonum* Extract and Low-Intensity Ultrasound on Motility, Viability, and DNA Fragmentation of Human Frozen-Thawed Semen Samples

**DOI:** 10.22074/ijfs.2020.5896

**Published:** 2020-07-15

**Authors:** Hamid Ekrami, Mansoureh Movahedin, Fereshteh Koosha, Zohreh Mazaheri, Manijhe Mokhtari-Dizaji

**Affiliations:** 1 Department of Anatomical Sciences, Faculty of Medical Sciences, Tarbiat Modares University, Tehran, Iran; 2 Basic Medical Science Research Center, Histogenotech Company, Tehran, Iran; 3 Department of Radiology Technology, Faculty of Allied Medical Sciences, Shahid Beheshti University of Medical Sciences, Tehran, Iran; 4 Department of Medical Physics, Faculty of Medical Sciences, Tarbiat Modares University, Tehran, Iran

**Keywords:** Antioxidants, *Calligonum*, Cryopreservation, Low-Intensity Ultrasound, Spermatozoa

## Abstract

**Background:**

The study aimed to evaluate the impact of *Calligonum* extract and US radiation on sperm parameters of cryopreserved human semen samples.

**Materials and Methods:**

In this experimental study, twenty-five semen specimens were obtained from healthy semen donors and incubated in human tubal fluid (HTF) medium supplemented with 10% human serum albumin (HSA) for 45 minutes. Samples were treated with *Calligonum* extract (10 μg/ml) alone (CGM group) and US radiation (LIPUS- exposed group) alone or a combination of both treatments (CGM+LIPUS). The US group received US stimulation (in both continuous and pulsed wave modes) at a frequency of 1 MHZ and intensity of 200 mW/cm2 for 200 seconds. Sperm morphology was assessed by Diff-Quik staining. The DNA fragmentation was evaluated the Halo sperm kit. Sperm parameters was analyzed by a computer-assisted semen analysis system. Reactive oxygen species (ROS) was assessed by flow cytometry.

**Results:**

The results showed that the treatment with *Calligonum* extract significantly (P<0.05) increased the progressive motility of spermatozoa in the CGM group as compared with the control group. The application of low-intensity US significantly (P<0.05) decreased the motility and viability of spermatozoa in the US group when compared with the control group. Our findings also indicated that the use of both low-intensity US in continuous mode and *Calligonum* extract slightly increased progressive motility; however, such an increase was not statistically significant. The rate of DNA fragmentation was considerably higher (P<0.05) in control and LIPUS-exposed groups than the other groups.

**Conclusion:**

Treatment of spermatozoa with *Calligonum* extract slightly improved the sperm parameters due to its antioxidant activity, on the other hand, according to our results, US radiation did not improve sperm parameters which may be due to interference with the motility of sperm, as well as its physical effects on spermatozoa.

## Introduction

Cryopreservation of human semen is considered one of the most vital and essential strategies for the preservation and maintenance of spermatozoa, and it is broadly applied in malignancies or other therapies which could damage to the functionality of testicles (1). During the cryopreservation process, the viability, motility and the integrity of chromatin might be adversely influenced, usually accompanied by the elevation of oxidative stress and excessive production of reactive oxygen species (ROS) (2). In the presence of increased ROS production, free radical oxygen molecules can target and attack to bis-allylic methylene groups of phospholipid-bound polyunsaturated fatty acids which are present in the plasma membrane of spermatozoa, leading to lipid peroxidation (3). The impact of lipid peroxidation on the sperm function include irreversible loss of motility, discharge of intracellular enzymes, sperm DNA damage, impairment of oocyte penetration and sperm-oocyte fusion, and apoptosis of spermatozoa in frozen media (4).

Many efforts have been made to minimize the rate of cryodamage in frozen spermatozoa during the cryopreservation process. For instance, the addition of antioxidant agents or cryoprotectants to the extender is one of the promising tools for the increase of sperm quality during the freeze-thaw process (5-7). Various types of antioxidants, such as vitamin C or E (8), genistein (5), and resveratrol (9) have been employed for cryopreservation. However, the yield of the cryopreservation procedure for human spermatozoa is not yet satisfactory, and new strategies are still warranted to improve the fidelity of cryopreservation. *Calligonum* *comosum* *(C. comosum)* is a medicinal plant abundantly found in the Egyptian desert, containing numerous polyphenol antioxidant agents. The beneficial roles of *C. comosum* may stem from its antioxidant activity, which has been extensively assessed *in vitro* (10). Some studies have demonstrated some biological properties of *C. comosum* extract on rodent models, such as the anti-inflammatory and anti-gastric ulcer activities (11).

Additionally, *C. comosum* showed the anticancer potential in mice inoculated with Ehrlich ascites carcinoma cells (12). It has been implicated that low-intensity pulsed ultrasound (LIPUS) at the incident power density from 0.5 to 3000 mW/cm^2^ have been used for bone healing (13). LIPUS can promote the growth of human skin fibroblasts, thereby the activation of the integrin receptors, RhoA(Ras homolog gene family, member A)/ ROCK, and Src-ERK signaling pathways (14, 15). The biological action of LIPUS occurs when the mechanical wave is converted into a biochemical signal within the cell as the mechanoreceptors and integrin are thought to be involved in this process. A number of studies have highlighted the formation of focal adhesions on the surface of the cells treated with LIPUS, which is mediated by the activation of integrin-associated signaling pathways (16, 17). Focal adhesions are large multi-protein complexes that could serve as a transmembrane bridge between the extracellular matrix (ECM) and the actin cytoskeleton. They could be identified in specific sites within the cell where clustered integrin receptors can interact with the ECM components on the outside and with the actin cytoskeleton on the inside of the cells. One of the main components of focal adhesion proteins involved in the transduction of the LIPUS signal from a mechanical force to a chemical messenger is focal adhesion kinase (FAK), which is phosphorylated when the cells are exposed to LIPUS (17, 18).

Hence, regarding the above statements, it would be plausible that LIPUS can enhance the penetration of herb extraction into the sperm via an increase in motility of spermatozoa. Therefore, the primary goal of the present study was to determine the impact of *C. comosum* extract alone, LIPUS signal, and the combination of both on the count, viability, total motility, progressive motility, DNA fragmentation, and morphology of spermatozoa during the freeze-thawing process.

## Materials and Methods

### Study design

In this experimental study, we evaluated the effects of *Calligonum* extract and LIPUS at a frequency of 1 MHZ (in pulsed and continues wave modes) on the cryopreservation cryopreservation of human spermatozoa. After the preparation of semen samples, each sample was liquated into 5 parts included; washed spermatozoa (control group), frozen-thawed spermatozoa, frozen-thawed spermatozoa treated with *Calligonum* extract at a concentration of 10 

μg/ml (CGM group), frozen-thawed spermatozoa exposed to the US radiation (LIPUS-exposed group) at a duty cycle of 40% (pulsed mode, at a frequency of 1 MHz, at the incident power density of 200 mW/cm2 for 200 seconds), and frozen-thawed spermatozoa treated with the combination of Calligonum extract and the US radiation with continues mode (CGM+LIPUS group). The present research was approved by Ethical Committee of Tarbiat Modares University (No. 52/6757 dated 30.11.92).

### Herb extraction

### The identification of the herb

The plant (*Calligonum* *comosum* L.) was collected by Dr. Hosein Batooli from the desert located at the proximity of Kashan, Iran, (33.9850°N, 51.4100°E). The taxonomic identity of the collected plant was confirmed by Dr. Abdoalrasool Haghir Ebrahim Abadi, Faculty of Science, Kashan University, Iran. Freshly collected plant materials were air-dried in the shade at room temperature. The aerial parts (stem, flowers, and leaves) of this herb were used for further investigations.

### The extraction protocol

Two kilograms of the fresh aerial parts of the plant (equal to 50% of the weight of a wildly-growing plant) were air-dried in the shade at room temperature, grounded, and exhaustively extracted by cold maceration with aqueous methanol (70%). The extract was evaporated under reduced pressure at 40°C to yield 80 g residue. The residue was suspended in distilled water and successfully fractionated with n-hexane, CH2Cl2, EtOAc, and n-BuOH (Thermo Fisher, USA) saturated with H_2_O. Each extract was evaporated under reduced pressure to yield 3, 7, 12 and 22 g residues, respectively.

### The antioxidant activity

The activity of free radical-scavenging of the methanolic extract of C. comosum was determined concerning the potential to neutralize the free radical-producing [2,2-diphenyl- 1-picrylhydrazyl (DPPH)] according to a method, as described previously (19, 20). Concisely, the level of DPPH was calculated by the measurement of the absorbance at the wavelength of 517 nm prior to and after the addition of a specific amount of the herb extract. Afterward, the inhibitory percentage of radical formation was estimated using the following equation: % inhibition= (A _control_ – A _sample_)/A _control_ ×100, where A _control_ is considered the absorbance of DPPH alone and A _sampl_e is regarded as the absorbance of DPPH in combination with the C. comosum extract.

### Semen sample collection

In this experimental study, semen samples were obtained from twenty-five fertile men with the average age of 34 (range, 25-50 years) who referred to the *in vitro* fertilization (IVF) center of Gandhi Hospital according to the World Health Organization (WHO) criteria. The process of semen collection was carried out by masturbation into a sterile, wide-mouthed, calibrated glass container after five days of sexual abstinence. The collected specimens were allowed to liquefy at 37°C for 30 minutes prior to the semen analysis. The entire samples were collected from individuals who referred for medical procedures during assisted reproductive technology (ART). We used the remaining samples obtained from patients, with their permission, for research purposes.

### Sperm preparation by the swim-up method

A fraction of motile spermatozoa was selected to be analyzed by the swim-up method. For this purpose, 1 ml of each semen sample (kept at 37°C and 5% CO_2_) was added to 3 ml of human tubal fluid (HTF, Genocell Co., Iran) supplemented with 10% human albumin serum (HAS, Biotest, Germany) and centrifuged at 2000 ×g for 3 minutes. Afterward, 0.5 ml of HTF supplemented with 10% HAS was gently added on the resultant pellets. The samples were then incubated at 37°C, 5% CO_2_, and inclined at a 45° angle to incubator for 45 minutes. Consequently, 0.5 ml of the uppermost medium was recovered, and the swim-up method was performed (21).

### Sperm freezing and thawing

Each processed semen sample was cryopreserved according to the standard protocol for sperm freezing. According to Li et al. (7) and Ibrahim et al. (22), semen samples were gently mixed with an equal volume of modified cryoprotective medium (Global Media, USA) supplemented with 10 μg/ml of *Calligonum* extract. The samples were kept at 4°C for 30 minutes and then frozen by placing the straws horizontally at 10 cm above the surface of liquid nitrogen (nitrogen vapor) at -80°C for 20 minutes. Finally, all frozen straws were stored in liquid nitrogen until use.

At the thawing stage, the cryo-straws were removed from liquid nitrogen and immediately immersed in a water bath at 37°C for at least 1-2 minutes. The thawed straws containing semen samples were flicked and inverted to mix the contents before sampling thoroughly and then washed with a culture medium HTF supplemented with 10% HAS (The samples were then treated with *Calligonum* extract (10 μg/ml)). At the end step, semen samples were centrifuged at 2000 ×g for 3 minutes to remove any trace of cryoprotectant in the freezing medium. The samples were analyzed for motility, viability, morphology, and DNA fragmentation (9, 23).

### Preparation of ultrasound device

The US device (Physiomed, Germany) was set up at the frequency of 1 MHz, incident power density of 200 mW/cm^2^, 200-second time period, and a 45-minute period as the duration of the experiment. These fine-tuned parameters were chosen based on our previous study published in this regard (24). The US stimulation was carried ou in which the transducer was put at the focal distance (3.5 cm) of a cell culture plate incubated in 5% CO_2_ at 32˚C. It was transmitted through the bottom of the well via coupling gel between the transducer and the tube. The device was transmitted through the bottom of the well via coupling the gel between the transducer and cell culture plate. Notably, no temperature change more than 1ºC was recorded during the US stimulation.

### Sperm stimulation using low-intensity pulsed ultrasound

Sperms maintained in the HTF medium supplemented with 10% HAS (Gibco, Germany). The samples were exposed to low-intensity pulsed ultrasound (LIPUS) (1 MHz, 200 mW/cm2 and 40% DC) alone or in combination with C. comosum coined as experimental groups. The control group was also cultured in the HTF medium supplemented with 10% HAS. After the US stimulation, spermatozoa were incubated for 45 minutes in 5% CO_2_ at 32˚C ,similar to other experimental groups. To investigate the sperm parameters, the mean number of whole cells per volume, viability, morphology, and motility were examined after the incubation process.

### Semen samples analysis

The sperm count, motility, morphology, viability, and DNA fragmentation were evaluated according to the guidelines introduced by the WHO (25). The sperm motility analysis was conducted using light microscopy (at ×400 magnification), combined with a computer-assisted semen analysis system (CASA, Video Test Sperm 1.2, Russia). A Makler chamber was used for the scoring of the sperm motility at room temperature. A minimum of 100 spermatozoa from at least five different fields was analyzed from each aliquot. Sperm morphology was assessed by the Diff-Quik staining method according to WHO guidelines by light microscopy (Labomed, USA). The sperm viability was determined by Eosin B (5% in saline) (Merck, Germany) staining technique at onestep. In this procedure, viable or dead spermatozoa are recognized by white (unstained) and pink (or red) coloration in the head region of the sperm cells, respectively.

### Sperm DNA fragmentation

The Halotech DNA G2 kit (Spain) was used to study the DNA fragmentation in frozen-thawed spermatozoa. Based on the sperm chromatin dispersion test (SCD), the intact frozen-thawed spermatozoa were diluted in a culture medium HTF supplemented with 10% HAS to achieve sperm concentration of 15-20 million per milliliter. In this method, 50 μl of the semen sample was added to 100μl of dissolved agarose (0.7%) (Sigma, US). Afterward, 25 μl of the cell suspension was transferred to slides, pre-coated with 0.65% agarose and covered with a coverslip without any air bubbles, and then incubated at 4°C for 5 minutes. After the removal of coverslips, the slides were horizontally immersed in a tray with freshly prepared acid denaturation solution (0.08 N HCl) for 7 minutes at 22°C in a dark place to create restricted single- stranded DNA from DNA breaks. The slides were then immersed in lysis Solution I [0.4 M Tris (Sigma, USA), 0.4 M 2-Mercaptophenol (Sigma, USA), 1% sodium dodecyl sulfate (SDS, Sigma, USA), and 50 mM Ethylenediaminetetraacetic acid (EDTA, Sigma, USA), pH=7.5 for 20 minutes and lysis Solution II (0.4 M Tris, 2 M NaCl, and 1% SDS, pH=7.5) for 5 minutes to remove nuclear proteins. Slides were then rinsed with distilled water for 5 minutes, followed by dehydration through an ascending gradient of ethanol (70, 90, and 100%) for 2 minutes. The slides were then placed at room temperature to be air-dried.

For Diff-Quik staining, the slides were incubated in eosin solution for 6 min; then, transferred into Azur B solution, for another 6 minutes. The nucleoid of spermatozoa with fragmented DNA did not develop a dispersion halo, or the halo was minimal. From each slide, a minimum of 500 spermatozoa was scored under an oil-immersion objective (×100 magnification) by light microscopy (Labomed, USA). The sperm cells showing no halo, small halo, medium halo, large halo, or fragmentation were separately scored. Spermatozoa indicating no halo/fragmentation were considered to have damaged chromatin, and the results were expressed as a percentage of sperm cells with DNA damage.

### Intracellular reactive oxygen species level measurement

Spermatozoa were rinsed with phosphate-buffered saline (PBS, Atocel, Austria) and incubated with 20 μM 2, 7- dichlorofluorescein diacetate (DCFH-DA, Life Technologies, USA), diluted in serum-free medium at 37°C for 45 minutes. The intracellular ROS level was immediately analyzed with flow cytometry at an excitation wavelength of 488 nm and an emission wavelength of 530 nm (26).

### Statistical analysis

The analysis of the values obtained in this study was performed by the SPSS version 19 (SPSS Inc., IBM company, USA), while the level of significance was set at P<0.001. The difference between the values of each group was analyzed by one-way analysis of variance (one-way ANOVA), followed by Tukey’s test. The data were expressed as the means ± standard deviation (means ± SD).

## Results

### The activity of radical scavenging of DPPH

The *C. comosum* extract showed free radical-scavenging activity in a dose-dependent manner when examined by the DPPH assay. The inhibitory concentration (IC_50_) value (the concentration of a given substance causing 50% inhibition of the DPPH) of the herb extract was 29.2 μg/ml when compared with ascorbic acid (positive control).

### Effect of *Calligonum* extract and/or LIPUS on sperm parameters

### Motility assessment

The demographic characteristics of patients are demonstrated in Table 1.

**Table 1:** The demography of semen samples


Characteristics	Average	SD

Age (Y)	34	3.041
Total motility (%)	80.7795	1.69
Grade A (%)	33.1496	1.74
Grade B (%)	30.7443	1.56
Grade C (%)	16.8856	1.39
Grade D (%)	19.4599	1.58
Grade A+B (%)	64.0651	2.08
Viability (%)	87	1.27
Normal morphology (%)	28.6397	4.26
SDF (%)	10.63	2.15
ROS (%)	11.0804	2.16


Grade A; Linear progressive motility, Grade B; Progressive motility, Grade C; Non-progressive motility, Grade D; Immotile, SDF; Sperm DNA fragmentation, ROS; Reactive oxygen species, and SD; Standard Diviation.

All of the sperm parameters, including viability, motility, and morphology in all experimental groups, are shown in Table 2. Accordingly, the percentage of total motility of spermatozoa in the fresh group was 89.37 ± 3.74, while this value was 81.30 ± 5.72 in the frozen-thawed group. After the addition of 10 μg/ml of Calligonum extract in the freezing medium increased the total motility; yet, such an increase was not statistically significant when compared with the control group (P≥0.05). LIPUS (in pulsed and continues mode waves) alone decreased the motility of spermatozoa, compared with the control group (P≤0.038), but the combination of *Calligonum* extract and LIPUS increased the motility to (81.90 ± 3.93) and (81.65 ± 5.18) respectively when used in pulsed mode and continuous modes waves. Also, there was an improvement in progressive motility of spermatozoa treated with the combination of *Calligonum* extract and LIPUS, compared with the frozen- thawed group; however, the difference between two groups was not statistically significant (P≥0.05).

### Viability assessment

As shown in Table 2, the viability of spermatozoa in all groups undergone the freeze-thawing process was significantly reduced as compared with the fresh group (P≤0.026). Such a reduction was more pronounced in the LIPUS-exposed and CGM-treated groups, compared with other groups. There was no significant difference between the CGM+LIPUS and control groups (frozen-thawed spermatozoa) (frozen-thawed spermatozoa, P≥0.05).

**Table 2:** Comparison of sperm parameters (± SD) between the experimental groups after frozen-thawed and treatment with 10 μg/ml Calligonum (CGM) extract and LIPUS (pulsed mode and continues wave)


GroupsSperm parameters	Control	Frozen -thawed	Frozenthawed+CGMextract	Frozenthawed+continueswave of ultrasound	Frozenthawed+continueswave oftrasound+CGMextract	Frozenthawed+pulsedmode ofultrasound	Frozenthawed+pulsedmode of ultrasound +CGMextract

Viability (%)	94.17^c^ ± 5.97	87.52 ± 4.75	90.11 ± 6.12	82.76^b^ ± 5.68	86.70 ± 7.12	81.82^b^ ± 2.87	87.94 ± 4.25
Total motility(%)	89.37 ± 3.74	81.30 ± 3.74	85.42 ± 4.28	76.76^ab^ ± 5.29	81.65 ± 5.18	75.86^ab^ ± 3.47	81.90 ± 3.93
Progressivemotility (%)	38.80 ± 2.57	34.81a ± 3.28	37.81 ± 4.13	33.04^ab^ ± 2.98	37.24 ± 5.11	32.60^ab^ ± 3.58	36.18^a^ ± 4.78
Normalmorphology (%)	30.11 ± 3.16	26.58 ± 3.54	28.41 ± 4.27	27.58 ± 3.95	30.82 ± 2.75	28.35 ± 4.82	30.05 ± 3.66


^a^; Significant difference with control group in the same row (P≤0.05), ^b^; Significant difference with freeze and thawed group + CGM extract, in the same row P≤0.026, and ^c^; Significant difference with other groups in the same row (P≤0.047).

**Table 3:** Assessment of fragmented DNA and free radicals percentage in all experimental groups


Groups	Control	Frozen- thawed	Frozen- thawed +CGM extract	Frozen- thawed +continues wave ofultrasound	Frozen- thawed +continues wave ofultrasound+CGMextract	Frozen- thawed +pulsed mode ofultrasound	Frozen- thawed +pulsed mode ofultrasound+CGMextract

Fragmented DNA(%) ± SD	24.5 ± 7.23	46.5^a^ ± 9.26	37 ± 4.29	38 ± 5.37	34 ± 5.17	51^a^ ± 11.85	42.5^a^ ± 7.39
Freeradicals(ROS)(%) ± SD	-	12.46 ± 7.06	9.18 ± 2.57	12.52 ± 8.14	7.77 ± 2.06	14.75 ± 4.67	10.05 ± 1.68


CGM; Calligonum extract, ROS; Reactive oxygen species, and ^a^; significant difference with control group in the same row (P≤0.042).

### Morphology assessment

According to Table 2, the normal morphology score was 30.11 ± 3.16 in the fresh group, while the normal morphology score was 26.58 ± 3.54 i\n the control group. The statistical analysis revealed that there was no significant difference in the score of normal morphology among all treated groups, namely, the CGM-treated, LIPUS-exposed, and CGM+LIPUS groups.

### Effect of Calligonum extract and/or LIPUS (pulsed or continuous mode waves) on DNA fragmentation

Table 3 shows the rate of DNA fragmentation in all experimental groups. The percentage of spermatozoa undergone DNA fragmentation was considerably higher in all groups than the fresh group. The rate of DNA fragmentation was significantly (P≤0.042) elevated in the frozenthawed (46.5 ± 9.26) and LIPUS-exposed groups (51 ± 11.85) in comparison with the control group.

### Effect of Calligonum extract and/or LIPUS on reactive oxygen species level

According to Table 3, the level of ROS in the control group receiving no treatment was 12.46 ± 7.06, while the level of ROS was decreased to 9.18 ± 2.57 in the CGM group. The statistical analysis demonstrated that the difference in the amount of ROS was not significant in all experimental groups as compared with the control group (P≥0.05).

## Discussion

Cryopreservation of human semen provides a valuable therapeutic opportunity for the management of patients who are at risk of infertility (27). However, during cryopreservation, spermatozoa experience physical and chemical stress that could result in detrimental changes in lipid composition of the cell membrane, leading to the excessive amount of ROS production, as well as a decrease in sperm motility and viability (28, 29). Notably, the osmotic stress and the alterations in the temperature may cause mechanical stress to the cell membrane of spermatozoa. Therefore, the changes mentioned above could reduce the fertilization capability of human spermatozoa after the cryopreservation process (30).

It has been implicated that antioxidant therapy can increase the quality of cryopreserved spermatozoa when employed both* in vitro* and *in vivo* (31). Numerous antioxidant agents have been indicated to have beneficial roles in the protection against cellular damages caused by cryopreservation-induced ROS, affecting sperm motility and viability. Hence, the application of antioxidant compounds to neutralize the deleterious impacts of oxidative damage would be of asset to improve the sperm parameters (32). Therefore, in this study, we examined the effects of *Calligonum* extraction, as an antioxidant agent, to annul the harmful effects of free radical molecules generated during the freeze-thawing process. We also applied LIPUS (in continuous and pulsed mode waves) to induce physical stimulation to spermatozoa for possible inciting the motility and viability of sperm cells after cryopreservation.

Our data revealed *Calligonum* extract at a concentration of 10 μg/ml slightly increased the sperm motility and viability, whereas it decreased DNA fragmentation and ROS level in human spermatozoa. The combination therapy using LIPUS and *Calligonum* extract could enhance sperm parameters when compared with the LIPUS-exposed group. Our findings were consistent with the previous studies performed in this area. For instance, Martinez- Soto et al. (29) evaluated the effects of extender supplemented with genistein on frozen-thawed human spermatozoa. They found that genistein, known as isoflavone compound, had antioxidant properties on cryopreserved spermatozoa. Their results also showed that the ROS production was decreased and the sperm motility was slightly improved in response to treatment with genistein. In another study, Banihani et al. (33) found that L-carnitine had positive effects on the improvement of sperm motility and viability during cryopreservation but had no effect on the reduction of DNA oxidation. Regarding our results, the addition of *Calligonum* extract to the freezing medium led to a significant increase in progressive motility.

On the other hand, LIPUS at the frequency of 1 MHZ and incident power density of 200 mW/cm^2^, in both pulsed and continuous mode waves, had adverse impacts on the sperm parameters. In the LIPUS-exposed group, the viability, as well as total and progressive motility was decreased while the number of none-motile spermatozoa was increased as compared with the fresh group. The US radiation alone increased intracellular ROS level and disrupted the balance of pro-oxidant and antioxidant contents in human spermatozoa, led to the elevated rate of DNA fragmentation. Also, our data demonstrated that LIPUS did not alter the morphology of frozen spermatozoa; however, the combinatory treatment of spermatozoa with *Calligonum* extract and LIPUS was capable of enhancing the sperm parameters during cryopreservation.

Previous studies have highlighted that LIPUS, as mechanical energy, could have therapeutic effects on bone and wound healing (34, 35). Also, it has been reported that it could expedite the process of tissue repair by the stimulation of the proliferation of fibroblasts and osteoblasts (17, 36). Furthermore, Xu et al. (37) have shown that LIPUS can stimulate the viability of freeze-thawed spermatozoa and it can increase the proliferation and differentiation of hematopoietic stem cells, obtained from fresh and cryopreserved peripheral blood leukapheresis product. However, inconsistent with other studies, our findings failed to show the beneficial effects of this therapeutic approach on the sperm parameters.

In the present study, the adverse effects of LIPUS on the sperm parameters may be due to the changes in the frequency of waves as a result of the sperm motility, leading to the reduction in the effectiveness of LIPUS penetration. Since the distribution of the US field is not homogenous homogenous, and it is susceptible to the reflection and attenuation once the field passes through the boundary separating two different media (38), the potency of US waves might be mitigated. So further examinations on applying other techniques for irradiating ultra sound on motile sperms is needed.

## Conclusion

Our results showed that *Calligonum* extract, at a concentration of 10 μg/ml, slightly enhanced the cellular parameters of cryopreserved spermatozoa diminished the rate of DNA fragmentation, and decreased the intracellular ROS level. Contrary to our expectation, LIPUS, in both pulsed and continuous mode waves, had the adverse effects on the sperm parameters which may stem from alterations in the temperature of the medium as a result of LIPUS treatment. It should be noted that the sperm motility might influence the frequency of the US waves, lowering the effectiveness of LIPUS treatment. This phenomenon can weaken the impact of LIPUS on the sperm parameters during cryopreservation.
